# Liver Transplant for Unusually Large Polycystic Liver Disease: Challenges and Pitfalls

**DOI:** 10.1155/2018/4863187

**Published:** 2018-02-01

**Authors:** Pablo Serrano Rodriguez, Alfred Sidney Barritt IV, David Allen Gerber, Chirag Sureshchandra Desai

**Affiliations:** ^1^Department of Surgery, Division of Abdominal Transplant Surgery, The University of North Carolina at Chapel Hill, Chapel Hill, NC 27599, USA; ^2^Division of Gastroenterology and Hepatology, The University of North Carolina at Chapel Hill, Chapel Hill, NC 27599, USA

## Abstract

Patients with polycystic liver disease are described in the literature as both recipient and donor for liver transplant. Due to well-preserved liver function, it is often difficult for these patients to receive an organ. Livers of these patients are often large and heavier than a normal organ. We describe two cases who had exceedingly large livers, weighing 14 and 19 kg. To the best of our knowledge and search, these are some of the heaviest explanted livers, and one of the patients incidentally received a liver from a donor with ADPKD. The aim of this report is to discuss the challenges and pitfalls of evaluating and listing, technical aspect of the transplant, possibility of transplanting a liver from a donor with a genetic cystic disease to a cystic disease recipient, and the related literature with some highlights on the facts from UNOS/OPTN data.

## 1. Introduction

Polycystic liver disease (PLD) can be associated with autosomal dominant polycystic kidney disease (ADPKD) and autosomal dominant polycystic liver disease (ADPLD) [1–3]. These are progressive diseases that can have an increase in liver size of up to 3.7% annually [4]. Patients can have cysts at an early age but normally do not have renal and/or hepatic manifestations until the 5th-6th decades of life. With large cysts, quality of life is affected due to either compressive symptoms or end organ damage that can progress to death [5, 6]. The medical management includes avoiding exogenous estrogen, medications like somatostatin analogues like octreotide, and mTOR inhibitors with variable success [3, 7, 8]. Invasive options include aspiration sclerotherapy, laparoscopic fenestration, and liver resection [9–14].

Of all patients diagnosed with PLD, 60% present with symptoms, 42% of the patients require at least one treatment of any kind, 23% require more than 1 intervention, 17% present with complications, and 8% of the patients fail to respond to any of these therapies. Patients with progressive disease with failed therapies either become overtly decompensated due to comorbidities or progress to end stage liver disease requiring orthotopic liver transplant (OLT) [15]. In most patients, model for end stage liver disease (MELD) score remains low. As MELD is the metric for liver graft allocation in the United States, these patients rarely receive priority for transplant. Since 2013, after the establishment of the share 35 rule, regional sharing of donor organs has increased, making it much harder for lower MELD score patients to have an opportunity for transplant. Some patients with PLD have associated renal impairment making their MELD score more competitive, but in the large majority, their score is still lower than other cirrhotic patients [16]. For that reason in 2015 the Organ Procurement and Transplantation Network (OPTN) published guidelines to standardize the allocation of exception points for patients with PLD. In order to be eligible for exception points, the patient needs to demonstrate hepatic decompensation, hemodialysis, or “compensated comorbidities” [17], rather than quality of life issue [18]. Patients transplanted for PCLD have 30-day morbidity of 41% and mortality of 5%. The 1- and 5-year survival of liver transplant alone are 93 and 91%. In patients receiving a simultaneous liver-kidney transplant, 1- and 5-year survival are lower, 86 and 80%, respectively [19], and 6% of patients required retransplantation. A majority of patients experience improvement in quality of life [20].

The large livers increase the technical difficulty of these procedures. We describe two cases of 51 and 98 kg women who had unusually large livers, weighing 14 and 19 kg, respectively. To the best of our knowledge and literature search, these are some of the heaviest explanted livers. Incidentally, one of our patients received a liver from a donor with ADPKD with multiple small liver cysts. The aim of reporting this series is to discuss the challenges and pitfalls of evaluating and listing this complex patient, the technical aspect of the transplant, and utility of transplanting a liver from one cystic disease donor to a cystic disease recipient while discussing the related literature.

## 2. Case Reports

**Figure 1:**
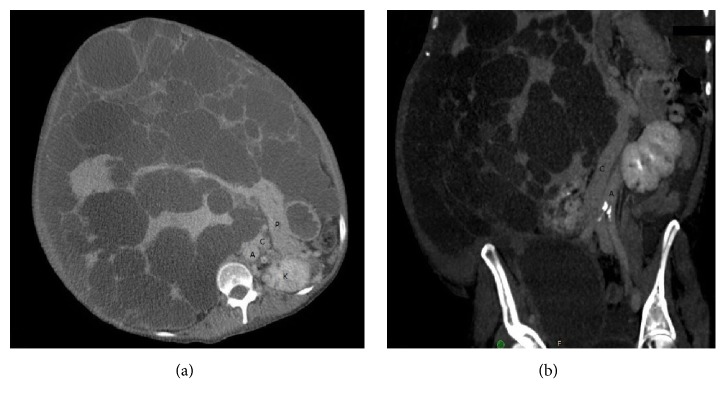
(a) Axial section CT showing abdominal anatomy with kidney (K) and pancreas (P) in relation to liver. (b) Coronal plane CT venogram showing vena cava (C) pushed on the left side of the aorta (A).

A 57-year-old female [177 cm (5′10) 51 kg (113)] with ADPKD had an extensive history of comorbidities, including ascites with multiple paracenteses, repeated spontaneous bacterial peritonitis, pyrosis, hyporexia, early satiety, abdominal pain, severe weight loss, and malnutrition. In the year prior to the transplant, she clinically deteriorated and was unable to perform basic chores. She had portal hypertension with esophageal varices and thrombocytopenia. She was listed at that point with MELD score of 18. Since MRI failed to detail vascular patency and anatomy, because of large cysts, CT venogram was done as a preoperative workup, which showed displacement of the inferior vena cava pushing it to the left side of the aorta due to large caudate lobe cyst (Figure 1). Portal vein was stretched and patent and hepatic artery was normal. While listed, she developed a right common iliac deep venous thrombosis (DVT) due to external compression from her hepatic cysts requiring her to be on anticoagulation pushing MELD score to 25. A suitable organ was offered to her from the local organ procurement organization. Donor details are described in Table 1.

Of note, during the donor evaluation, ADPKD was diagnosed with multiple kidney cysts and with liver involvement including small-disseminated cysts but with almost normal size. During the transplant hepatectomy, the extremely enlarged liver was difficult to mobilize, and multiple cysts had to be transected in order to mobilize the organ from the abdominal cavity and obtain vascular control. Anatomy was distorted making the dissection of the portal vain and the inferior vena cava more complicated. We kept all vessels as long as possible. After explant the liver weighed 14 kg. A standard technique was followed with caval replacement without venovenous bypass (VVB). After the procedure, the patient was extubated on postoperative day one with downtrending LFTs. She was started on tacrolimus, mycofenolic acid, and a steroid taper. She was discharged home 10 days after the procedure without any complications. At 6-month follow-up, she has significant improvement in quality of life, increased appetite, and improved nutritional status.

The second case was a 53-year-old female [177 cm (5′10) 93 kg (205)] with history of ADPKD with similar symptoms as the first case; she had a hepatic hydrothorax that required monthly tapping to improve her shortness of breath. She had a native MELD of 10 with severe malnutrition. Exception points were obtained to increase to a MELD of 23. Donor details are in Table 1.

**Figure 2:**
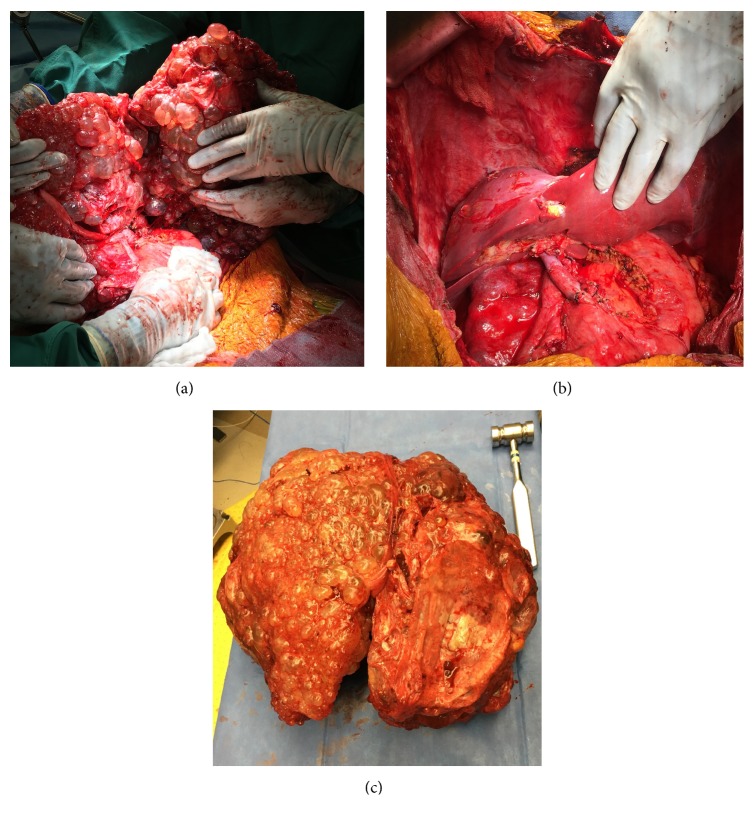
(a) Intraop. imaging of native liver. (b) Liver after reperfusion. (c) Explanted recipient liver.

During the transplant, 21 liters of ascites was removed. Transplant surgery was performed similar to the first case and the patient received the same immunosuppression as the previous one. Explant in this case weighed 19.4 kg (Figure 2). Of note, during the explant, when we started cutting the suprahepatic cava, after clamping vessels, and cutting PV and infrahepatic IVC, due to the weight of the liver, RHV area tore and got retracted in the clamp. We applied another clamp above after dissecting the diaphragm to get adequate cuff for the anastomosis. Postoperative course was significant for ascites and hydrothorax requiring prolonged weaning and tracheostomy. She had an episode of rejection, which was successfully treated. After a month long stay, she has been discharged to rehab.

## 3. Discussion

Data from OPTN/UNOS from February 2002 to December 2015 show that from 117,427 patients listed, 620 (0.5%) were PLD cases. Of these cases, 351 (56.6%) had transplants and 81 (13.1%) died on the waitlist or became too sick to transplant. The average wait time was 196 days, that is, longer than the wait time of patients with HCC (130 days) and patients with chronic liver failure (58 days). Of all PLD patients listed, 269 (43.4%) received exception points, making them more likely to have transplant compared with those who were listed without exception points (78.8% versus 39.6%). Their waitlist mortality and removal from the list were significantly higher than those of HCC though lower than those of CLF (21.4% versus 5.2% versus 26.9%, resp.) [21]. Looking at these numbers, it is highly intuitive that exception petitions should be filed for these patients and listing be considered very early on.

The sheer size of the liver can make it difficult to evaluate patency of the portal vein and vena cava. As many centers perform MRI for pretransplant evaluation of liver disease, it is noteworthy that the multiple cysts make it difficult to assess vascular patency and anatomy. In contrast, a triple-phase CT scan and reconstructed venogram were very useful in both of our cases. Nutritional status of these patients is also a concern, thereby requiring these patients to be on either enteral or parenteral nutritional support preoperatively.

During the surgery, the typical cirrhotic liver is small and can be easily manipulated. Enlarged polycystic livers make mobilization and dissection of the hilum more complicated. The typical liver volume is 1.1 kg, and the average polycystic liver increases to 6.695 kg [22]. Mobilization and visualization improves with fenestration of select cysts, and this has to be performed carefully to avoid injuring the displaced vessels and collaterals. Most of the vessels are under tension making them susceptible to tearing and rupture. The IVC is frequently displaced and compressed in 60% of patients [22]. During the hepatectomy, it is imperative to preserve vascular length to compensate for the size mismatch of the donor graft and avoid tension during implantation. Due to such a large size, it is preferable to do standard bicaval replacement technique over the piggyback technique for the transplant. These patients have portal hypertension, and with modern anesthetic management, they usually do not require venovenous bypass (VVB). One problem we had encountered as mentioned earlier is weight dragging the suprahepatic cava and causing retraction. In hindsight, maybe in such a heavy liver, to prevent this drag, we should cut the suprahepatic cava before the infrahepatic, so that natural tendency while cutting top cava to pull on the liver is avoided.

Due to the lack of availability of suitable donor liver grafts, there is increasing pressure to accept more unconventional donors for the purpose of transplantation [23]. Patients with PLD have been described in the literature as donors, for both living and cadaveric donation [24, 25]. Following Gigot's classification, type 1 includes the presence of less than 10 large hepatic cysts measuring no more than 10 cm in maximum diameter [24] and can be suitable for transplantation as a donor. It would be interesting to evaluate strategies to prevent enlargement of cyst from donor, but current literature does not support any evidence-based measure. PLD patients can undergo transplant from living donors, but this may be suitable only in few cases. Development of postsurgical ascites is a challenge after any form of surgery for PLD. However, with good graft function, improved nutrition, and gentle diuretics, most can be controlled.

In conclusion, patients with PLD are more complicated and disadvantaged. They often require exception point petition and the utilization of innovative donors. It is essential to avoid undue tension on small vessels during the hepatectomy and be aware of the distorted anatomy preoperatively. Size and weight of the liver are the most challenging parts of this surgery, but with appropriate precautions, good outcomes can be obtained.

## Figures and Tables

**Table 1:** Demographics and lab work for both donors showing terminal value and peak value in parenthesis.

	Donor 1	Donor 2
Age (years)	32	21
Race	African American	Caucasian
Gender	Male	Female
AST	25 (83)	48 (84)
ALT	20 (58)	17 (25)
Alkaline phosphatase	53 (60)	35 (49)
Bilirubin	0.3 (0.4)	4 (4)
Creatinine	0.74 (1.65)	0.9 (1.7)
INR	0.9 (1.1)	1.4 (1.6)
Albumin	3.6 (2.1)	3.1 (3.8)
Sodium	155 (158)	134 (155)
Potassium	4 (5.1)	4 (4.1)
Hemoglobin	7.7 (13.3)	10.5 (10.5)
Platelet	246 (383)	88 (164)
Vasopressors	No	No

## Abbreviations

ADPKD: Autosomal dominant polycystic kidney disease
ADPLD: Autosomal dominant polycystic liver disease
CLF: Chronic liver failure
HA: Hepatic artery
IVC: Inferior vena cava
MELD: Model for end stage liver disease
OLT: Orthotopic liver transplant
PLD: Polycystic liver disease
PV: Portal vein
RHV: Right hepatic vein.
